# Chiropractic students call for action against unsubstantiated claims

**DOI:** 10.1186/s12998-020-00318-5

**Published:** 2020-05-13

**Authors:** Joshua Plener, Ben Csiernik, Geronimo Bejarano, Jesper Hjertstrand, Benjamin Goodall

**Affiliations:** 1 Canadian Memorial Chiropractic College, 6100 Leslie St., Toronto, ON M2H 3J1 Canada; 2 Palmer College of Chiropractic, Florida Campus, 4777 City Center Pkwy, Port Orange, FL 32129 USA; 3 University of South Wales, Treforest Campus, Llantwit Road, Pontypridd, CF37 1DL UK; 4 AECC University College, Parkwood Campus, Bournemouth, England BH5 2DF UK

**Keywords:** Chiropractic, Regulation, Student, Education, Public safety

## Abstract

**Background:**

The 2019 coronavirus pandemic is a current global health crisis. Many chiropractic institutions, associations, and researchers have stepped up at a time of need. However, a subset of the chiropractic profession has claimed that spinal manipulative therapy (SMT) is clinically effective in improving one’s immunity, despite the lack of supporting scientific evidence. These unsubstantiated claims contradict official public health policy reflecting poorly on the profession. The aim of this commentary is to provide our perspective on the claims regarding SMT and clinically relevant immunity enhancement, drawing attention to the damaging ramifications these claims might have on our profession’s reputation.

**Main text:**

The World Federation of Chiropractic released a rapid review demonstrating the lack of clinically relevant evidence regarding SMT and immunity enhancement. The current claims contradicting this review carry significant potential risk to patients. Furthermore, as a result of these misleading claims, significant media attention and public critiques of the profession are being made. We believe inaction by regulatory bodies will lead to confusion among the public and other healthcare providers, unfortunately damaging the profession’s reputation. The resulting effect on the reputation of the profession is greatly concerning to us, as students.

**Conclusion:**

It is our hope that all regulatory bodies will protect the public by taking appropriate action against chiropractors making unfounded claims contradicting public health policy. We believe it is the responsibility of all stakeholders in the chiropractic profession to ensure this is carried out and the standard of care is raised. We call on current chiropractors to ensure a viable profession exists moving forward.

## Background

Chiropractic students are frequently reminded that it has never been a better time to be a chiropractor [1]. We the authors are excited at the prospect of joining the profession and making a difference, as we agree the future of chiropractic looks bright. However, we are questioning the above sentiment in light of the current global crisis of Coronavirus disease 2019 (COVID-19). The world is struggling to come to terms with COVID-19, chiropractic students are no exception. The delivery of education has changed, many campus clinics have closed, and future plans of graduating students have been placed in jeopardy. Despite these trying times, we appreciate and are encouraged by the initiative and collaborative spirit of the chiropractic profession. Chiropractic institutions have ensured the availability of quality education in an online format [2], associations have supported and advocated for their members [3], and researchers have appraised literature to assist with knowledge translation [4].

In spite of the above-mentioned efforts, a small minority of the profession has publicly claimed that chiropractic treatments have a role to play in the fight against COVID-19. Specifically, this minority of chiropractors, best characterized by the International Chiropractors Association (ICA) statement, have made unsubstantiated assertions that spinal manipulative therapy (SMT) can augment immune function to improve health outcomes [5]. Bearing this in mind, the goal of this commentary is to provide our perspective on the claims regarding SMT and clinically relevant immunity enhancement, drawing attention to the damaging ramifications these claims might have on our profession’s reputation.

## Main text

Shortly after the World Health Organization (WHO) declared COVID-19 a pandemic [6], the World Federation of Chiropractic (WFC), which supports the WHO on this matter, released a rapid review on March 19th, 2020 [4]. The review, which examined cited material claiming support for the effectiveness of SMT and immunity enhancement, concluded that “[there is] no credible, scientific evidence that spinal adjustment/manipulation has any clinically relevant effect on the immune system …” [4]. As students, we are not only encouraged by the WFC’s response, but support their strong and swift action in exposing unsubstantiated claims contradicting official public health policy. In addition, we are pleased with the numerous national chiropractic associations that released statements in support of the WFC’s conclusions [7–11]. However, as mentioned above, a minority of chiropractors are contradicting the WFC’s review, spreading the belief that SMT can clinically enhance the body’s immune response. This is deeply concerning to us, as these chiropractors are making unsupported claims while in positions of power and influence over trusting patients. Of even greater concern, these claims can lead to fatal consequences as a result of potential exposure while in the clinical environment. Fomites, such as furniture, treatment tables, and examination equipment, have the potential to create an optimal environment for virus transmission. Furthermore, the chiropractic profession has a very high score (96/100) pertaining to “physical proximity to others” [12], making social/physical distancing impossible. The benefits of any chiropractic treatment provided during COVID-19 must out-weigh the potential risk of spreading the virus to patients. At a time when social/physical distancing is required to ‘flatten the curve’, only emergency/acute care visits with the appropriate personal protective equipment should be carried out. The chiropractic profession is not in a position to continue non-emergent in-person visits, while still placing public health interests first.

### The need for evidence-based education

The aforementioned unsubstantiated claims conflict with the education received at many chiropractic institutions. We are proud of the institutions that have signed the International Chiropractic Education Collaboration Position Statement (ICECPS), clearly demonstrating their desire for an evidence-based approach to teaching and healthcare [13]. For us, it is disappointing that only two North American chiropractic programs have signed this position statement. At a time when scientifically unsupported claims are being made, we call for all chiropractic institutions to adopt the ICECPS position. We also believe the Councils on Chiropractic Education (CCE), who are the regulators and accreditors of chiropractic programs [14], need to participate in this effort. The CCE-International, which is recognized by the WHO as an information source, was established in 2003 to provide guidance to the CCEs [15]. Though the CCE-International supports an evidence-based musculoskeletal (MSK) model, this sentiment is not held by all CCEs [15, 16]. We are disappointed that some accredited schools continue to instruct non-evidence-based teachings [15–17]. Therefore, we urge all CCEs to take the necessary steps to ensure an evidence-based curriculum is universally adopted.

### Reputational damage

In some countries, chiropractors fall below other medical professionals such as nurses and physicians, with respect to honesty and ethical standards [18]. As a result, significant reputational damage can follow when unfounded claims are made that undermine public health policy. Disappointingly, the increased media attention we have seen leads us to believe the damage has already begun. In a recent article published by the Canadian Broadcasting Corporation (CBC) on March 30th, 2020, the claims made by some chiropractors regarding SMT and immunity were said to “muddy the waters” and add unnecessary confusion to the public [19]. Similar news stories have been written by Fox6 in the state of Wisconsin and CBC in British Columbia [20, 21]. As a result of this media attention, we are deeply worried for future ramifications if these claims continue. Gíslason states “ongoing resistance to aligning with the scientific literature has been seen by some as the central issue in impeding professional legitimization, scientific and societal support and inclusion within the wider healthcare professional landscape” [22]. We believe unsubstantiated claims made by a select few are generalized to the profession. This has cast a dark shadow on the profession in the eyes of critics and the healthcare community.

Scientists are speaking out against misinformation currently circulating around COVID-19 [23, 24]. As Berinsky points out, rumors are easy to identify, but the spread of false information is difficult to undo [25]. However, research has shown that misinformation corrected by the original source has demonstrated greater success in rectifying the public message [25, 26]. Therefore, in order to mitigate some of the reputational damage, we call on all chiropractors and organizations spreading misinformation to retract their statements and issue corrections. As we realize this is unlikely, we call on regulators to issue retractions to unsubstantiated statements chiropractors have made, in addition to their normal punitive action.

### Call for regulatory action

The unfounded claims regarding SMT and clinically relevant immunity enhancement is not only unsupported by evidence, but goes against standards set out by regulatory agencies [27–29]. We agree with the views of Marcon et al., that “it is hoped that the regulatory bodies will recognize these types of discourse [non-evidence-based claims] as problematic and will act accordingly” [30]. Inaction by regulatory bodies can lead to public confusion and negatively impact the image of the profession. If disciplinary actions are not taken by regulatory bodies, public perception will continue to be that all chiropractors agree with the false claims made by a small, yet vocal minority. This inaction will result in the continual spread of these unfounded beliefs [31]. We are concerned for the profession that we will be entering, as the profession’s reputation will be tarnished if swift action is not taken against these chiropractors. While we appreciate and support the regulatory bodies who have begun to take action against these dangerous claims, we call for further action and decisiveness. Similar to statements made by Leboeuf-Yde et al., we feel it is time for the profession to act before it is too late [31]. As students, we call not only on regulatory bodies to step up, but all stakeholders who have a vested interest in the profession to pursue their professional responsibility; reporting such behaviour in order to protect the public interest and assist with regulatory enforcement of the profession.

## Conclusions

The arguments expressed in this commentary are fueled by great concern, frustration, and embarrassment. We entered this profession wanting to make a difference in people’s lives, now we fear for what the state of the profession will be once we graduate. It is our hope that regulatory bodies will hold chiropractors who make unsupported and potentially harmful claims accountable for their actions. We call for a strong stance to be taken against these unsubstantiated claims and do not condone this unacceptable behaviour. As students, we are worried for the profession’s reputation and call on current chiropractors to ensure we have a viable profession moving forward.

## Data Availability

Not applicable.

## Abbreviations

COVID-19: Coronavirus disease 2019
ICA: International Chiropractors Association
SMT: Spinal Manipulative Therapy
WHO: World Health Organization
WFC: World Federation of Chiropractic
ICECPS: International Chiropractic Education Collaboration Position Statement
MSK: Musculoskeletal
CCE: Councils on Chiropractic Education
CBC: Canadian Broadcasting Corporation

## References

[1] Kawchuk G. A wonderful @ECUConventions this in Budapest with #chiropractic colleagues this weekend. Some word to leave you with 2018. https://twitter.com/GNK1/status/1001040665780867072. Accessed 12 Apr 2020.

[2] Canadian Memorial Chiropractic College. Regarding the coronavirus – COVID-19. 2020. https://www.cmcc.ca/covid19updates. Accessed 12 Apr 2020.

[3] British Chiropractic Association. BCA joins forces with over 80, 000 Healthcare Professionals to raise concerns about lack of support for businesses and the self-employed effected by COVID-19. 2020. https://chiropractic-uk.co.uk/bca-joins-forces-80000-healthcare-professionals-raise-concerns-lack-support-businesses-self-employed-effected-covid-19/. Accessed 12 Apr 2020.

[4] Kawchuk G, Goertz C, Axén I, Descarreaux M, French S, Haas M (2020). The effect of spinal adjustment/manipulation on immunity and the immune system: a rapid review of relevant literature.

[5] International Chiropractors Association. Immune function and chiropractic; What does the evidence provide?. 2020. http://www.chiropractic.org/wp-content/uploads/2020/03/Updated-Report-of-3-28-wtih-fixed-biblio.pdf. Accessed 12 Apr 2020.

[6] Verity R, Okell L, Dorigatti I, Winskill P, Whittaker C, Imai N (2020). Estimates of the severity of coronavirus disease 2019; a model-based analysis. Lancet.

[7] AHPRA. False and misleading advertising on COVID-19. 2020. https://www.chiropracticboard.gov.au/News/2020-03-31-false-and-misleading-advertising-on-covid-19.aspx?fbclid=IwAR05rSI8BGO8eFNyloieWybU_U-arp1Wit1ABWJTj6Hln6fu13Q3D1zTSSE. Accessed 12 Apr 2020.

[8] Statement by the chiropractic profession. 2020. https://chiropractic-uk.co.uk/wp-content/uploads/2020/03/Statement-by-the-Chiropractic-Profession-21-March-2020.pdf. Accessed 12 Apr 2020.

[9] General Chiropractic Council. 2020. Claims made about the effect of chiropractic treatment on COVID-19. https://www.gcc-uk.org/news/entry/claims-made-about-the-effect-of-chiropractic-treatment-on-covid-19. Accessed 12 Apr 2020.

[10] Canadian Chiropractic Association. Canada’s Chiropractors, Patient Safety and COVID-19. 2020. https://www.chiropractic.ca/blog/canadas-chiropractors-patient-safety-and-covid-19/. Accessed 12 Apr 2020.

[11] American Chiropractic Association. Let’s work together to protect and serve our patients, staff, families and communities. 2020. https://www.acatoday.org/News-Publications/Publications/ACA-Blogs/ArtMID/6925/ArticleID/1551/Let%E2%80%99s-Work-Together-to-Protect-and-Serve-Our-Patients-Staff-Families-and-Communities. Accessed 12 Apr 2020.

[12] Gamio L. The workers who face the greatest coronavirus risk. In: New York Times. 2020. https://www.nytimes.com/interactive/2020/03/15/business/economy/coronavirus-worker-risk.html?referringSource=articleShare&fbclid=IwAR3_ruNEIMjxb0Z63m2fCbOqxPc4_x5a_KkmOk9ey3-k0Gh5BJboJXLjIuU. Accessed 12 Apr 2020.

[13] The International Chiropractic Education Collaboration; Clinical and Professional Chiropractic Education: A Position Statement. https://fnks.org/wp-content/uploads/2020/01/Edu-position-statement-w.-SU-300120-1.docx. Accessed 12 Apr 2020.

[14] Innes SI, Leboeuf-Yde C, Walker BF (2016). How comprehensively is evidence-based practice represented in councils on chiropractic education (CCE) educational standards: a systematic audit. Chiropr Man Therap..

[15] Innes SI, Leboeuf-Yde C, Walker BF (2018). Comparing the old to the new: A comparison of similarities and differences of the accreditation standards of the chiropractic council on education-international from 2010 to 2016. Chiropr Man Therap.

[16] Innes SI, Leboeuf-Yde C, Walker BF (2019). A failed review of CCE site inspection standards and processes. Chiropr Man Therap..

[17] Puhl AA, Reinhart CJ, Doan JB, McGregor M, Injeyan HS (2014). Relationship between chiropractic teaching institutions and practice characteristics among Canadian doctors of chiropractic: a random sample survey. J Manip Physiol Ther.

[18] Saad L. Nurses top list of most honest and ethical professions. In: Gallup News Service. 2006. https://news.gallup.com/poll/25888/nurses-top-list-most-honest-ethical-professions.aspx. Accessed 12 Apr 2020.

[19] Bellemare A, Ho J, Nicholson K. Chiropractors told to remove posts claiming their methods boost immune system and prevent COVID-19. In: Canadian Broadcasting Corporation. 2020. https://www.cbc.ca/news/health/chiropractors-immune-system-covid-19-1.5511008. Accessed 12 Apr 2020.

[20] Polcyn B. Can a spinal adjustment protect you from COVID-19? Associations call Mequon doc’s claim ‘misleading’. In: Fox6. 2020. https://fox6now.com/2020/04/02/can-a-spinal-adjustment-protect-you-from-covid-19-associations-call-mequon-doctors-claim-misleading/. Accessed 12 Apr 2020.

[21] Lindsay, B. B.C. Chiropractors warned about ‘inappropriate’ claims on COVID-19. In: Canadian Broadcasting Corporation. 2020. https://www.cbc.ca/news/canada/british-columbia/bc-chiropractors-covid-19-1.5497922?fbclid=IwAR2PXyMNCjcrCCd-iDdPRZ8F61rnHaIlxAcTI1rbhucK8jfHjXMvIaShvIQ. Accessed 12 Apr 2020.

[22] Gíslason HF, Salminen JK, Sandhaugen L, Storbråten AS, Versloot R, Roug I (2019). The shape of chiropractic in Europe: a cross sectional survey of chiropractor's beliefs and practice. Chiropr Man Therap.

[23] Mian A, Khan S (2020). Coronavirus: the spread of misinformation. BMC Med.

[24] Calisher C, Carroll D, Colwell R, Corley R, Daszak P, Drosten C (2020). Statement in support of the scientists, public health professionals, and medical professionals of China combatting COVID-19. Lancet..

[25] Berinsky AJ (2017). Rumors and health care reform: experiments in political misinformation. Br J Polit Sci.

[26] Walter N, Tukachinsky R (2020). A meta-analytic examination of the continued influence of misinformation in the face of correction: how powerful is it, why does it happen, and how to stop it?. Commun Res.

[27] General Chiropractic Council. The Code. 2016. https://www.gcc-uk.org/chiropractic-standards/the-code?fbclid=IwAR2U1sVWMw1nFOJUMyuH4vBSsRZ3ZGLpRHn-fRkUvMuhCOJsmmsd52ChEMs. Accessed 12 Apr 2020.

[28] AHPRA. Code of conduct. 2019. https://www.chiropracticboard.gov.au/Codes-guidelines/Code-of-conduct.aspx?fbclid=IwAR0DNIXMsRrfKIEvko9mFuKy3UQNfZaYtaFzXmPbe5DKqD0zUYvLPeOaQeA. Accessed 12 Apr 2020.

[29] College of Chiropractors of Ontario. Standard of Practice S-016: Advertising. 2019. https://www.cco.on.ca/wp-content/uploads/2020/02/S-016.pdf. Accessed 12 Apr 2020.

[30] Marcon AR, Murdoch B, Caulfield T (2019). The “subluxation” issue: an analysis of chiropractic clinic websites. Arch Physiother.

[31] Leboeuf-Yde C, Innes SI, Young KJ, Kawchuk GN, Hartvigsen J (2019). Chiropractic, one big unhappy family: better together or apart?. Chiropr Man Therap..

